# Is It Necessary to Cross the Cervicothoracic Junction in Posterior Cervical Decompression and Fusion for Multilevel Degenerative Cervical Spine Disease? A Systematic Review and Meta-Analysis

**DOI:** 10.3390/jcm12082806

**Published:** 2023-04-11

**Authors:** Honghao Yang, Jixuan Huang, Yong Hai, Zhexuan Fan, Yiqi Zhang, Peng Yin, Jincai Yang

**Affiliations:** Department of Orthopedic Surgery, Beijing Chao-Yang Hospital, Capital Medical University, Gongti South Rd, No. 8, Beijing 100020, China

**Keywords:** posterior cervical fusion, degenerative cervical spine disease, adjacent segment disease, reoperation rate, meta-analysis

## Abstract

Background: Posterior cervical decompression and fusion (PCF) is a common procedure for treating patients with multilevel degenerative cervical spine disease. The selection of lower instrumented vertebra (LIV) relative to the cervicothoracic junction (CTJ) remains controversial. This study aimed to compare the outcomes of PCF construct terminating at the lower cervical spine and crossing the CTJ. Methods: A comprehensive literature search was performed for relevant studies in the PubMed, EMBASE, Web of Science, and Cochrane Library database. Complications, rate of reoperation, surgical data, patient-reported outcomes (PROs), and radiographic outcomes were compared between PCF construct terminating at or above C7 (cervical group) and at or below T1 (thoracic group) in patients with multilevel degenerative cervical spine disease. A subgroup analysis based on surgical techniques and indications was performed. Results: Fifteen retrospective cohort studies comprising 2071 patients (1163 in the cervical group and 908 in the thoracic group) were included. The cervical group was associated with a lower incidence of wound-related complications (RR, 0.58; 95% CI 0.36 to 0.92, *p* = 0.022; 831 patients in cervical group vs. 692 patients in thoracic group), a lower reoperation rate for wound-related complications (RR, 0.55; 95% CI 0.32 to 0.96, *p* = 0.034; 768 vs. 624 patients), and less neck pain at the final follow-up (WMD, −0.58; 95% CI −0.93 to −0.23, *p* = 0.001; 327 vs. 268 patients). However the cervical group also developed a higher incidence of overall adjacent segment disease (ASD, including distal ASD and proximal ASD) (RR, 1.87; 95% CI 1.27 to 2.76, *p* = 0.001; 1079 vs. 860 patients), distal ASD (RR, 2.18; 95% CI 1.36 to 3.51, *p* = 0.001; 642 vs. 555 patients), overall hardware failure (including hardware failure of LIV and hardware failure occurring at other instrumented vertebra) (RR, 1.48; 95% CI 1.02 to 2.15, *p* = 0.040; 614 vs. 451 patients), and hardware failure of LIV (RR, 1.89; 95% CI 1.21 to 2.95, *p* = 0.005; 380 vs. 339 patients). The operating time was reasonably shorter (WMD, −43.47; 95% CI −59.42 to −27.52, *p* < 0.001; 611 vs. 570 patients) and the estimated blood loss was lower (WMD, −143.77; 95% CI −185.90 to −101.63, *p* < 0.001; 721 vs. 740 patients) when the PCF construct did not cross the CTJ. Conclusions: PCF construct crossing the CTJ was associated with a lower incidence of ASD and hardware failure but a higher incidence of wound-related complications and a small increase in qualitative neck pain, without difference in neck disability on the NDI. Based on the subgroup analysis for surgical techniques and indications, prophylactic crossing of the CTJ should be considered for patients with concurrent instability, ossification, deformity, or a combination of anterior approach surgeries as well. However, long-term follow-up outcomes and patient selection-related factors such as bone quality, frailty, and nutrition status should be addressed in further studies.

## 1. Introduction

Degenerative cervical spine disease, including myelopathy, radiculopathy, amyotrophy, deformity, pseudarthrosis, and ossification of the posterior longitudinal ligament (OPLL), has become one of the most common and debilitating spinal disorders, causing neck and arm pain, disability, and poor health-related quality of life (HRQoL) [1,2,3,4]. With an increasing proportion of aging individuals, the demand for surgical treatment has consistently increased in the last decade [5]. Among the surgical procedures, posterior cervical decompression and fusion (PCF) is commonly used for patients with multilevel degenerative cervical spine disease [6].

Surgery around the cervicothoracic junction (CTJ) is challenging because of its unique anatomical and biomechanical characteristics. The CTJ is the transition zone between the flexible lordotic cervical spine and rigid kyphotic thoracic spine [7]. Biomechanical research indicated that the selection of lower instrumented vertebra (LIV) of the PCF construct terminating at the CTJ would result in two adjacent rigid lever arms joined together with the mobile C7-T1 intervertebral disc in between [8]. These unphysiological biomechanics increase the intradiscal pressures and motion of C7-T1, which would accelerate the degeneration of this level and induce adjacent segment disease (ASD) or other mechanical complications. Therefore, some studies have recommended prophylactic extension of the fusion construct into the upper thoracic spine, crossing the CTJ, to reduce the rate of complications and need for reoperations [9,10,11]. In contrast, some investigators reported that this procedure would further increase surgical invasiveness and disrupt the posterior stabilising structures, which have no benefits in solid fusion [12,13,14].

Several meta-analyses have provided detailed evidence on this topic [15,16,17,18]. However, due to the limited number of studies, the literature has offered conflicting results and recommendations, and the selection of LIV relative to the CTJ remains controversial. Since new comparative studies with larger sample sizes have emerged in recent years, this systematic review and meta-analysis aimed to compare the mechanical complications, surgical complications, wound-related complications, systemic complications, reoperation rates, surgical data, patient-reported outcomes (PROs), and radiographic outcomes between PCF constructs terminating at the lower cervical spine and crossing the CTJ in patients with multilevel degenerative cervical spine disease.

## 2. Materials and Methods

This study was designed according to the Preferred Reporting Items for Systematic Reviews and Meta-Analyses (PRISMA) guidelines and registered with PROSPERO (ID: CRD42022375526) [19,20].

### 2.1. Search Strategy

PubMed, EMBASE, Web of Science, and Cochrane Library databases were searched using the following terms: (((posterior) AND (cervical)) AND ((fusion) OR (arthrodesis))) AND ((cervicothoracic) OR (thoracic)).

The literature search was updated on 13 November 2022. Two reviewers independently screened the titles and abstracts, and any differences were resolved by discussion with a third reviewer.

### 2.2. Inclusion and Exclusion Criteria

The inclusion criteria were as follows: (1) patients who underwent PCF (or combined anterior support) for degenerative cervical spine disease; (2) studies in which the lower instrumented vertebrae (LIV) of the PCF terminating at or above C7 (cervical group), not crossing the CTJ, were selected; (3) studies comparing patients with the LIV of the PCF terminating at or below T1 (thoracic group), crossing the CTJ; (4) studies with the following primary outcomes: mechanical complications, surgical complications, wound-related complications, systemic complications, and reoperations; and secondary outcomes: surgical data, patient-reported outcomes (PROs), and radiographic outcomes.

The exclusion criteria were as follows: (1) studies that included patients with trauma, infection, tumour, multiple sclerosis, or intradural pathology; (2) studies that reported the outcomes of PCF without subgroups based on the selection of LIV (cervical or thoracic); (3) reviews, case reports, and biomechanical research objectives; (4) studies with no available full text; (5) duplicate cohorts; and (6) non-English publications.

### 2.3. Assessment of Study Quality

The study quality was assessed independently by two reviewers using the Newcastle-Ottawa scale (NOS) recommended by the Cochrane Handbook version 5.2.0. The level of evidence rating was assigned according to published guidelines [21].

### 2.4. Outcomes

Mechanical complications included overall adjacent segment disease (ASD), overall hardware failure, pseudarthrosis, and distal junctional kyphosis (DJK). Overall, ASD includes distal ASD and proximal ASD. Overall hardware failure includes hardware failure of LIV and hardware failure occurring at other instrumented vertebrae, but only the data of hardware failure of LIV were collected in the current study. Surgical complications included epidural haematoma, dural tears, and neurologic deficits. Wound-related complications included wound dehiscence and surgical site infections. Reoperation was classified according to the aetiology, including mechanical, surgical, and wound-related complications. Reoperation included both the reoperation at the early period and late period after surgery. Surgical data included the operating time (ORT), estimated blood loss (EBL), and length of hospital stay (LOS). PROs included the Neck Disability Index (NDI), numeric rating scale (NRS) for neck pain (NP) and arm pain (AP), and Japanese Orthopaedic Association (JOA) scores at baseline and at the time of the final follow-up. Radiographic outcomes indicated C2-7 cervical lordosis (CL), C2-7 cervical sagittal vertical axis (cSVA), and T1 slope at baseline and at the final follow-up.

### 2.5. Data Extraction

Data extraction was performed independently by two reviewers. Demographic and clinical information including age, sex, body mass index (BMI), diagnosis, active smoking status, fusion levels, history of previous surgery, prior or concurrent anterior approach surgery, and follow-up duration were recorded. Data for the 15 clinical variables were extracted for the analysis. Continuous outcomes included ORT, EBL, LOS, NDI, NRS-NP, NRS-AP, JOA score, C2-7 CL, C2-7 cSVA, and T1 slope. Dichotomous outcomes included mechanical complications, surgical complications, wound-related complications, systemic complications, and reoperations.

### 2.6. Data Analysis

All statistical analyses were performed using the Stata version 15.1. Variables reported in at least two studies were analysed. For continuous outcomes, the weighted mean difference (WMD) was used to estimate the effect. The effect measure of dichotomous outcomes is displayed as a risk ratio (RR). The mean and standard deviation values of continuous outcomes or the counts and percentages of dichotomous outcomes for data-point comparisons are also displayed. Statistical heterogeneity among studies was evaluated using the I-square test and Cochran’s Q test. If the I^2^ value was less than 50% and the *p*-value was greater than 0.10, a fixed-effects model was used. If the I^2^ value was greater than 50% or the *p*-value was less than 0.10, we performed leave-one-out sensitivity analysis, which indicates excluding each study one by one and recalculating the pooled estimates on the remaining studies. If a source of potential heterogeneity could not be found, a random-effects model was used. Subgroup analysis was performed based on the surgical techniques (solely posterior or presence of anterior support) and indications for surgery (specific myelopathy/radiculopathy or general). The indications for surgery in the general subgroup were heterogeneous, including myelopathy, radiculopathy, amyotrophy, deformity, pseudarthrosis, and OPLL. To assess the impact of follow-up duration and age on each outcome, meta-regression analysis using a random-effects model was performed.

### 2.7. Assessment of Publication Bias

Potential publication bias was assessed by applying Egger’s test, and significance was considered when the *p*-value was less than 0.10 [22]. If publication bias was indicated, we further evaluated the number of missing studies by applying the “trim and fill” method and recalculated the pooled WMD or RR with the addition of those missing studies [23].

## 3. Results

### 3.1. Study Selection

**Figure 1:**
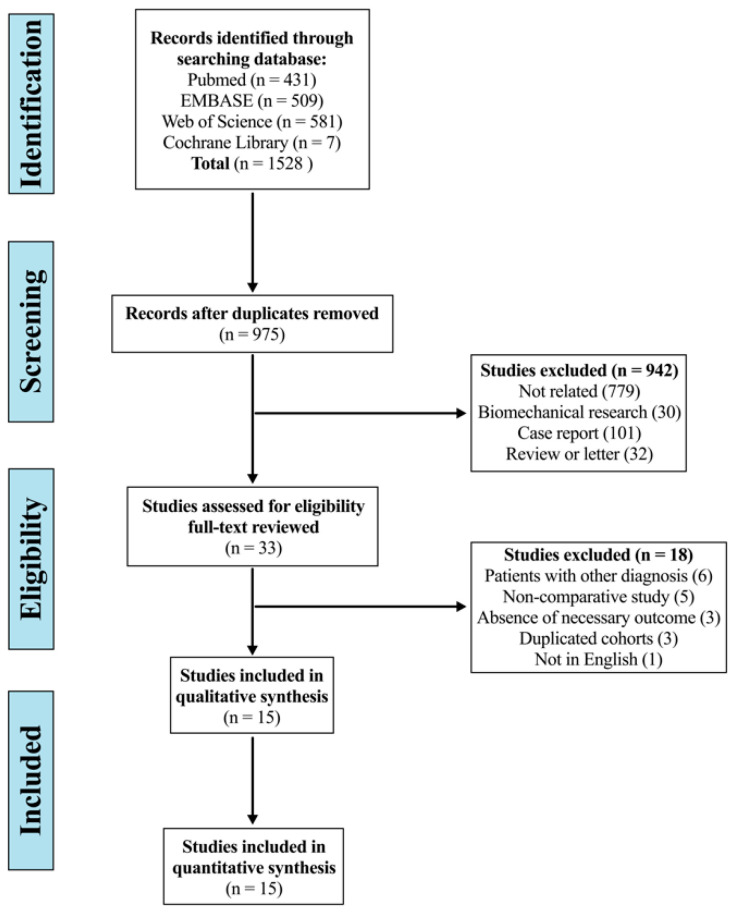
Flow diagram depicting the literature review, search strategy, and selection process.

The systematic search yielded 1528 articles, of which 553 were duplicates, and 942 were excluded by screening the title and abstract. After a full-text review, 18 studies were considered improper: six for including patients with diagnosis of trauma, infection, or tumour [13,14,24,25,26,27]; five for non-comparative study (cervical group vs. thoracic group) [9,28,29,30,31]; three for the absence of necessary outcomes [12,32,33]; three for partly duplicated cohorts [11,34,35]; and one for non-English study [36]. Finally, 15 studies were included in this systematic review and meta-analysis (Figure 1) [37,38,39,40,41,42,43,44,45,46,47,48,49,50,51].

### 3.2. Assessment of Study Quality and Publication Bias

The quality of the included studies was assessed using the Newcastle-Ottawa Scale (Table 1). All the studies were retrospective cohort studies. Of the 15 studies included, eight were of high quality with scores of 8–9, and seven were of moderate quality with scores of 6–7. The level of evidence was III in 14 studies and IV in one study. Publication bias was not detected.

### 3.3. Characteristics of Included Studies

Fifteen studies, comprising 2071 patients with degenerative cervical spine disease, were included. Of these patients, 1163 underwent PCF with LIV terminating at or above C7 and 908 patients with LIV terminating at or below T1. Characteristics of the included studies and patients are presented in Table S1. There were no significant differences at baseline between the two groups in the male-to-female ratio (1.36 vs. 1.16, *p* = 0.305), BMI (29.20 ± 6.13 kg/m^2^ vs. 28.92 ± 6.08 kg/m^2^, *p* = 0.202), active smoking status (20.1% vs. 25.0%, *p* = 0.260), NDI (31.33 ± 11.79 vs. 33.82 ± 11.24, *p* = 0.165), NRS-NP (5.39 ± 2.58 vs. 5.02 ± 2.88, *p* = 0.075), NRS-AP (5.48 ± 2.66 vs. 5.62 ± 2.67, *p* = 0.517), JOA score (10.94 ± 3.20 vs. 10.37 ± 2.98, *p* = 0.399), C2-7 CL (9.93° ± 10.06° vs. 8.48° ± 11.18°, *p* = 0.408), C2-7 cSVA (26.71 ± 13.13 mm vs. 29.07 ± 13.91 mm, *p* = 0.075), and T1 slope (24.72° ± 8.45° vs. 23.86° ± 9.08°, *p* = 0.333). In addition to the shorter fusion levels (4.28 ± 0.71 vs. 6.09 ± 1.24, *p* < 0.001), the cervical group was significantly younger (62.04 ± 10.53 years vs. 63.43 ± 10.51 years, *p* = 0.011) and had a lower proportion of prior or concurrent anterior support (17.0% vs. 26.2%, *p* < 0.001) compared with the thoracic group. The duration of follow-up was 34.68 ± 14.85 months in the cervical group and 30.08 ± 17.40 months in the thoracic group (*p* = 0.217).

### 3.4. Mechanical Complications

#### 3.4.1. Overall Adjacent Segment Disease

**Figure 2:**
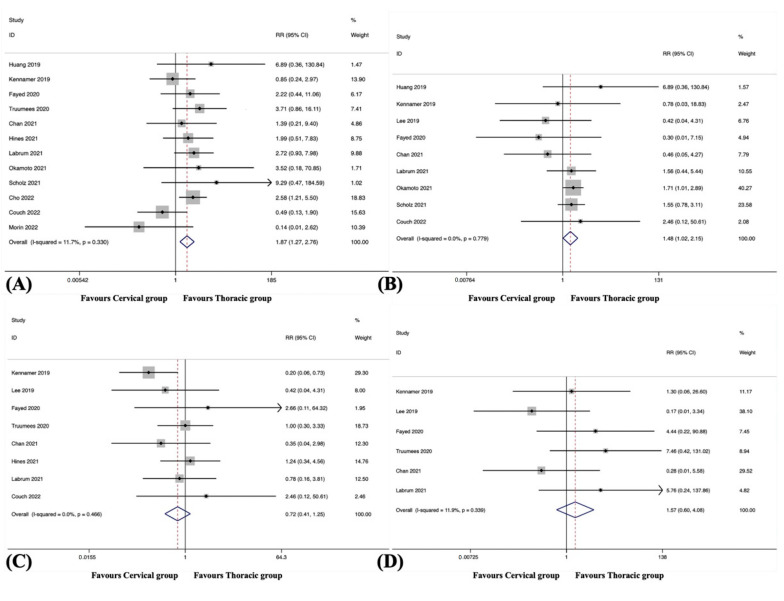
(**A**) Forest plot of overall adjacent segment disease [38,39,41,42,43,44,45,46,48,49,50,51]; (**B**) Forest plot of overall hardware failure [38,39,40,41,42,44,45,46,51]; (**C**) Forest plot of pseudarthrosis [39,40,41,42,43,44,48,51]; (**D**) Forest plot of distal junctional kyphosis [39,40,41,42,44,48].

Overall ASD was observed in 12 studies, including 1079 patients in the cervical group and 860 patients in the thoracic group [38,39,41,42,43,44,45,46,48,49,50,51]. The pooled results revealed a significantly higher incidence of overall ASD in the cervical group than in the thoracic group (RR, 1.87; 95% CI 1.27 to 2.76, *p* = 0.001), with no significant heterogeneity among studies (I^2^ = 11.7%, *p* = 0.330) (Table S2 and Figure 2A).

#### 3.4.2. Distal Adjacent Segment Disease

Distal ASD was observed in eight studies, including 642 patients in the cervical group and 555 patients in the thoracic group [38,42,44,46,48,49,50,51]. The pooled results revealed a significantly higher incidence of distal ASD in the cervical group than in the thoracic group (RR, 2.18; 95% CI 1.36 to 3.51, *p* = 0.001), with no significant heterogeneity among studies (I^2^ = 21.5%, *p* = 0.259).

#### 3.4.3. Proximal Adjacent Segment Disease

Proximal ASD was observed in four studies, including 382 patients in the cervical group and 298 patients in the thoracic group [38,42,49,51]. The pooled results revealed no significant difference in the incidence of proximal ASD between the groups (RR, 1.18; 95% CI 0.34 to 4.13, *p* = 0.799), with no substantial heterogeneity among studies (I^2^ = 0.0%, *p* = 0.667).

#### 3.4.4. Overall Hardware Failure

Overall hardware failure was observed in nine studies, including 614 patients in the cervical group and 451 patients in the thoracic group [38,39,40,41,42,44,45,46,51]. The pooled results revealed a significantly higher incidence of overall hardware failure in the cervical group than in the thoracic group (RR, 1.48; 95% CI 1.02 to 2.15, *p* = 0.040), with no substantial heterogeneity among studies (I^2^ = 0.0%, *p* = 0.779) (Figure 2B).

#### 3.4.5. Hardware Failure of LIV

Hardware failure of LIV was observed in six studies, including 380 patients in the cervical group and 339 patients in the thoracic group [38,42,44,45,46,51]. The pooled results revealed a significantly higher incidence of hardware failure of LIV in the cervical group than in the thoracic group (RR, 1.89; 95% CI 1.21 to 2.95, *p* = 0.005), with no substantial heterogeneity among studies (I^2^ = 0.0%, *p* = 0.737).

#### 3.4.6. Pseudarthrosis

Pseudarthrosis was observed in eight studies, including 839 patients in the cervical group and 599 patients in the thoracic group [39,40,41,42,43,44,48,51]. The pooled results revealed no significant difference in the incidence of pseudarthrosis between the groups (RR, 0.72; 95% CI 0.41 to 1.25, *p* = 0.237), with no substantial heterogeneity among studies (I^2^ = 0.0%, *p* = 0.466) (Figure 2C).

#### 3.4.7. Distal Junctional Kyphosis

DJK was observed in six studies, including 517 patients in the cervical group and 348 patients in the thoracic group [39,40,41,42,44,48]. The pooled results revealed no significant difference in the incidence of DJK between the groups (RR, 1.57; 95% CI 0.60 to 4.08, *p* = 0.358), with no significant heterogeneity among studies (I^2^ = 11.9%, *p* = 0.339) (Figure 2D).

### 3.5. Surgical Complications

#### 3.5.1. Overall

**Figure 3:**
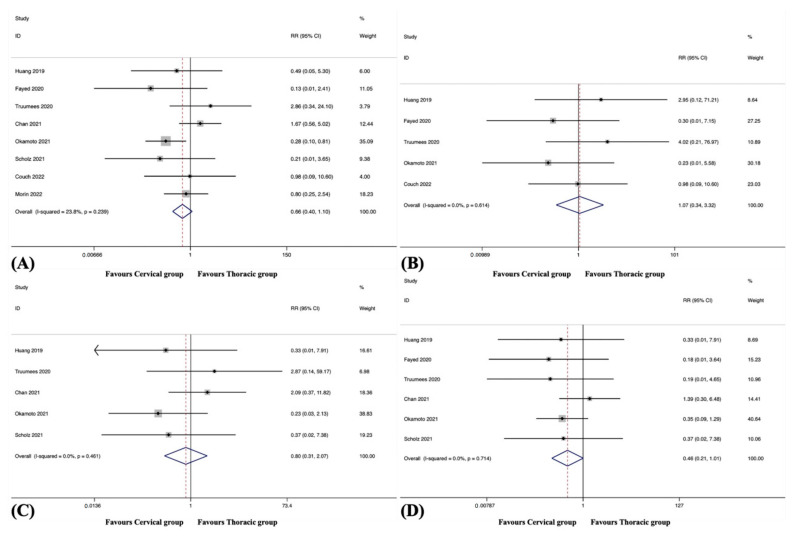
(**A**) Forest plot of overall surgical complication rate [38,41,42,45,46,48,49,51]; (**B**) Forest plot of epidural haematoma [38,42,45,48,51]; (**C**) Forest plot of dural tears [38,41,45,46,48]; (**D**) Forest plot of neurologic deficits [38,41,42,45,46,48].

Surgical complications were observed in eight studies, including 646 patients in the cervical group and 508 patients in the thoracic group [38,41,42,45,46,48,49,51]. The pooled results revealed no significant difference in the overall surgical complication rate between the groups (RR, 0.66; 95% CI 0.40 to 1.10, *p* = 0.113), with no significant heterogeneity among studies (I^2^ = 23.8%, *p* = 0.239) (Figure 3A).

#### 3.5.2. Epidural Haematoma

Epidural haematoma was observed in five studies, including 492 patients in the cervical group and 327 patients in the thoracic group [38,42,45,48,51]. The pooled results revealed no significant difference in the incidence of epidural haematoma between the groups (RR, 1.07; 95% CI 0.34 to 3.32, *p* = 0.907), with no substantial heterogeneity among studies (I^2^ = 0.0%, *p* = 0.614) (Figure 3B).

#### 3.5.3. Dural Tears

Dural tears were observed in five studies, including 329 patients in the cervical group and 274 patients in the thoracic group [38,41,45,46,48]. The pooled results revealed no significant difference in the incidence of dural tears between the groups (RR, 0.80; 95% CI 0.31 to 2.07, *p* = 0.647), with no substantial heterogeneity among studies (I^2^ = 0.0%, *p* = 0.461) (Figure 3C).

#### 3.5.4. Neurologic Deficits

Neurologic deficits were observed in six studies, including 408 patients in the cervical group and 344 patients in the thoracic group [38,41,42,45,46,48]. The pooled results revealed no significant difference in the incidence of neurologic deficits between the groups (RR, 0.46; 95% CI 0.21 to 1.01, *p* = 0.052), with no substantial heterogeneity among studies (I^2^ = 0.0%, *p* = 0.714) (Figure 3D).

#### 3.5.5. Wound-Related Complications

**Figure 4:**
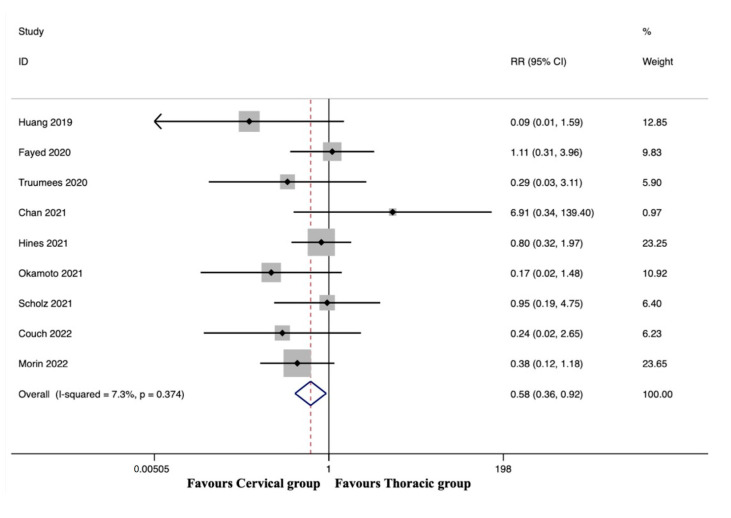
Forest plot of wound-related complications [38,41,42,43,45,46,48,49,51].

Wound-related complications were observed in nine studies, including 831 patients in the cervical group and 692 patients in the thoracic group [38,41,42,43,45,46,48,49,51]. The pooled results revealed a significantly lower incidence of wound-related complications in the cervical group than in the thoracic group (RR, 0.58; 95% CI 0.36 to 0.92, *p* = 0.022), with no significant heterogeneity among studies (I^2^ = 7.3%, *p* = 0.374) (Figure 4).

#### 3.5.6. Systemic Complications

Systemic complications were observed in four studies, including 161 patients in the cervical group and 178 patients in the thoracic group [38,41,45,46]. The pooled results revealed no significant difference in the incidence of systemic complications between the groups (RR, 0.24; 95% CI 0.05 to 1.10, *p* = 0.066), with no substantial heterogeneity among studies (I^2^ = 0.0%, *p* = 0.999).

### 3.6. Reoperation

#### 3.6.1. Overall

**Figure 5:**
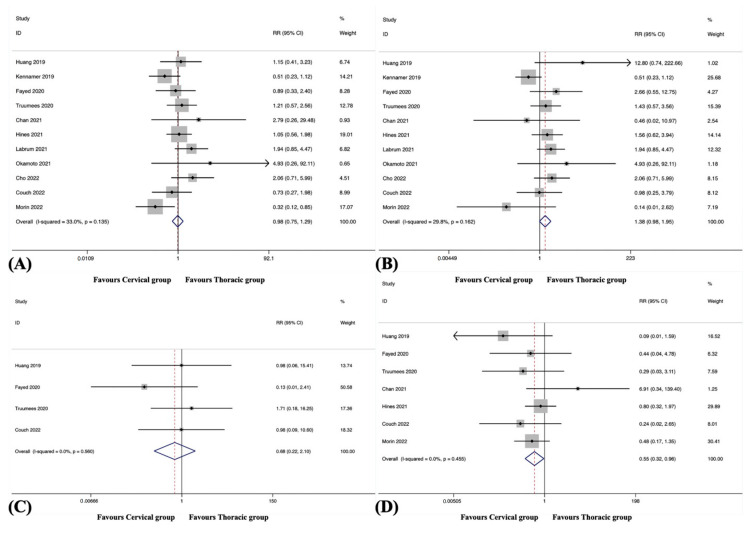
(**A**) Forest plot of overall reoperation rate [38,39,41,42,43,44,45,48,49,50,51]; (**B**) Forest plot of reoperation rate following mechanical complications [38,39,41,42,43,44,45,48,49,50,51]; (**C**) Forest plot of reoperation rate following surgical complications [38,42,48,51]; (**D**) Forest plot of reoperation rate following wound-related complications [38,41,42,43,48,49,51].

Reoperations were observed in 12 studies [38,39,41,42,43,44,45,46,48,49,50,51], and significant heterogeneity was detected (I^2^ = 52.9%, *p* = 0.016). A sensitivity analysis revealed that the source of heterogeneity was the study by Scholz et al. [46]. When this study was omitted, there was no significant heterogeneity (I^2^ = 33.0%, *p* = 0.135). The remaining 11 studies included 1059 patients in the cervical group and 822 patients in the thoracic group. The pooled results revealed no significant difference in the overall reoperation rate between the groups (RR, 0.98; 95% CI 0.75 to 1.29, *p* = 0.898) (Figure 5A).

#### 3.6.2. Reoperation Rate following Mechanical Complications

The reoperation rate following mechanical complications was observed in 12 studies [38,39,41,42,43,44,45,46,48,49,50,51], and significant heterogeneity was detected (I^2^ = 46.6%, *p* = 0.038). A sensitivity analysis revealed that the source of heterogeneity was the study by Scholz et al. [46]. When this study was omitted, there was no significant heterogeneity (I^2^ = 29.8%, *p* = 0.162). The remaining 11 studies included 1059 patients in the cervical group and 822 patients in the thoracic group. The pooled results revealed no significant difference in the reoperation rate following mechanical complications between the groups (RR, 1.38; 95% CI 0.98 to 1.95, *p* = 0.063) (Figure 5B).

#### 3.6.3. Reoperation Rate following Surgical Complications

The reoperation rate following surgical complications was observed in four studies, including 449 patients in the cervical group and 297 patients in the thoracic group [38,42,48,51]. The pooled results revealed no significant difference in the reoperation rate following surgical complications between the groups (RR, 0.68; 95% CI 0.22 to 2.10, *p* = 0.499), with no substantial heterogeneity among studies (I^2^ = 0.0%, *p* = 0.560) (Figure 5C).

#### 3.6.4. Reoperation Rate following Wound-Related Complications

The reoperation rate following wound-related complications was observed in seven studies, including 768 patients in the cervical group and 624 patients in the thoracic group [38,41,42,43,48,49,51]. The pooled results revealed a significantly lower reoperation rate following wound-related complications in the cervical group than in the thoracic group (RR, 0.55; 95% CI 0.32 to 0.96, *p* = 0.034), with no substantial heterogeneity among studies (I^2^ = 0.0%, *p* = 0.455) (Figure 5D).

### 3.7. Surgical Data

#### 3.7.1. Operating Time

**Figure 6:**
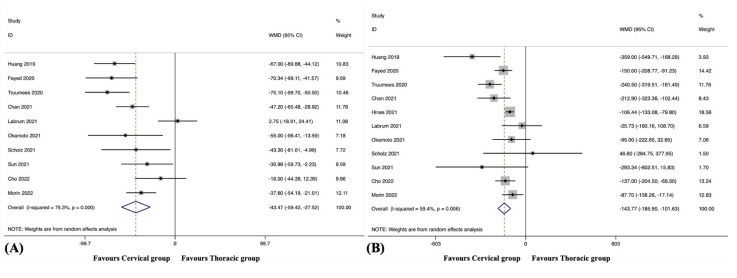
(**A**) Forest plot of operating time [38,41,42,44,45,46,47,48,49,50]; (**B**) Forest plot of estimated blood loss [38,41,42,43,44,45,46,47,48,49,50].

ORT was reported in 10 studies, including 611 patients in the cervical group and 570 patients in the thoracic group [38,41,42,44,45,46,47,48,49,50]. Significant heterogeneity was detected (I^2^ = 75.3%, *p* < 0.001). The pooled results revealed a significantly reduced ORT in the cervical group compared to the thoracic group (WMD, −43.47; 95% CI −59.42 to −27.52, *p* < 0.001) (Figure 6A).

#### 3.7.2. Estimated Blood Loss

EBL was reported in 11 studies, including 721 patients in the cervical group and 740 patients in the thoracic group [38,41,42,43,44,45,46,47,48,49,50]. Significant heterogeneity was detected (I^2^ =59.4%, *p* = 0.006). The pooled results revealed a significantly reduced EBL in the cervical group compared to the thoracic group (WMD, −143.77; 95% CI −185.90 to −101.63, *p* < 0.001) (Figure 6B).

#### 3.7.3. Length of Hospital Stay

LOS was recorded in seven studies, including 621 patients in the cervical group and 596 patients in the thoracic group [41,42,43,44,46,48,49]. Significant heterogeneity was detected (I^2^ =81.0%, *p* < 0.001). The pooled results revealed no significant difference in LOS between the groups (WMD, −0.40; 95% CI −1.28 to 0.48, *p* = 0.375).

### 3.8. Patient-Reported Outcomes

#### 3.8.1. Neck Disability Index

**Figure 7:**
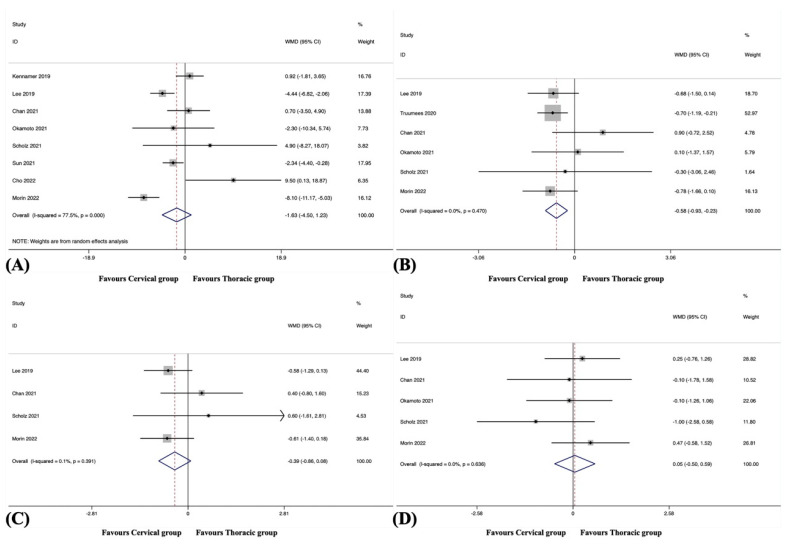
(**A**) Forest plot of Neck Disability Index score at the final follow-up [39,40,41,45,46,47,49,50]; (**B**) Forest plot of numeric rating scale for neck pain at the final follow-up [40,41,45,46,48,49]; (**C**) Forest plot of numeric rating scale for arm pain at the final follow-up [40,41,46,49]; (**D**) Forest plot of Japanese Orthopaedic Association score at the final follow-up [40,41,45,46,49].

The NDI score at the final follow-up was reported in eight studies, including 413 patients in the cervical group and 278 patients in the thoracic group [39,40,41,45,46,47,49,50]. Significant heterogeneity was detected (I^2^ = 77.5%, *p* < 0.001). The pooled results revealed no significant difference in the NDI score at the final follow-up between the groups (WMD, −1.63; 95% CI −4.50 to 1.23, *p* = 0.264) (Figure 7A).

#### 3.8.2. Numeric Rating Scale for Neck Pain

The NRS-NP score at the final follow-up was reported in six studies, including 327 patients in the cervical group and 268 patients in the thoracic group [40,41,45,46,48,49]. The pooled results revealed a significantly better NRS-NP score at the final follow-up in the cervical group than in the thoracic group (WMD, −0.58; 95% CI −0.93 to −0.23, *p* = 0.001), with no substantial heterogeneity among studies (I^2^ = 0.0%, *p* = 0.470) (Figure 7B).

#### 3.8.3. Numeric Rating Scale for Arm Pain

The NRS-AP score at the final follow-up was reported in four studies, including 128 patients in the cervical group and 151 patients in the thoracic group [40,41,46,49]. The pooled results revealed no significant difference in the NRS-AP score at the final follow-up between the groups (WMD, −0.39; 95% CI −0.86 to 0.08, *p* = 0.106), with no substantial heterogeneity among studies (I^2^ = 0.1%, *p* = 0.391) (Figure 7C).

#### 3.8.4. Japanese Orthopaedic Association Score

The JOA score at the final follow-up was reported in five studies, including 139 patients in the cervical group and 151 patients in the thoracic group [40,41,45,46,49]. The pooled results revealed no significant difference in the JOA score at the final follow-up between the groups (WMD, 0.05; 95% CI −0.50 to 0.59, *p* = 0.864), with no substantial heterogeneity among studies (I^2^ = 0.0%, *p* = 0.636) (Figure 7D).

### 3.9. Radiographic Outcome

#### 3.9.1. C2-7 Cervical Lordosis

**Figure 8:**
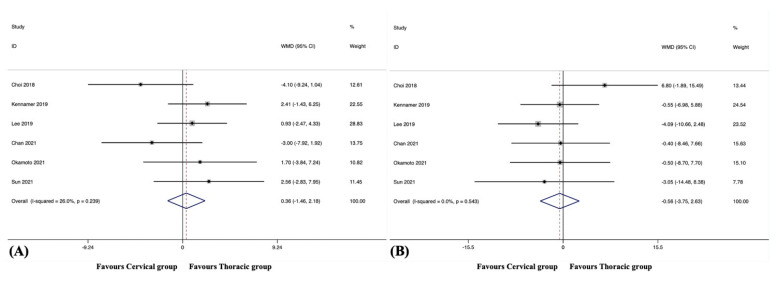
(**A**) Forest plot of C2-7 cervical lordosis at the final follow-up [37,39,40,41,45,47]; (**B**) Forest plot of C2-7 cervical sagittal vertical axis at the final follow-up [37,39,40,41,45,47].

The C2-7 CL at the final follow-up was reported in six studies, including 301 patients in the cervical group and 160 patients in the thoracic group [37,39,40,41,45,47]. The pooled results revealed no significant difference in C2-7 CL at the final follow-up between the groups (WMD, 0.36; 95% CI −1.46 to 2.18, *p* = 0.699), with no significant heterogeneity among studies (I^2^ = 26.0%, *p* = 0.239) (Figure 8A).

#### 3.9.2. C2-7 Cervical Sagittal Vertical Axis

The C2-7 cSVA at the final follow-up was reported in six studies, including 301 patients in the cervical group and 160 patients in the thoracic group [37,39,40,41,45,47]. The pooled results revealed no significant difference in the C2-7 cSVA at the final follow-up between the groups (WMD, −0.56; 95% CI −3.75 to 2.63, *p* = 0.731), with no substantial heterogeneity among studies (I^2^ = 0.0%, *p* = 0.543) (Figure 8B).

#### 3.9.3. T1 Slope

The T1 slope at the final follow-up was reported in three studies, including 101 patients in the cervical group and 97 patients in the thoracic group [40,41,45]. The pooled results revealed no significant difference in the T1 slope at the final follow-up between the groups (WMD, −1.94; 95% CI −4.72 to 0.83, *p* = 0.170), with no substantial heterogeneity among studies (I^2^ = 0.0%, *p* = 0.912).

### 3.10. Subgroup Analysis

#### 3.10.1. Subgroup Analysis Based on Surgical Techniques

When the posterior approach was used in isolation, the subgroup analysis revealed a significantly lower incidence of wound-related complications (RR, 0.52; 95% CI 0.29 to 0.93, *p* = 0.028), a shorter ORT (WMD, −38.29; 95% CI −57.84 to −18.75, *p* < 0.001), a lower EBL (WMD, −130.25; 95% CI −186.65 to −73.85, *p* < 0.001), and a better NDI score at the final follow-up (WMD, −3.60; 95% CI −6.35 to −0.85, *p* = 0.010), while it revealed a higher incidence of hardware failure of LIV (RR, 2.28; 95% CI 1.25 to 4.15, *p* = 0.007) and a higher reoperation rate for mechanical complications (RR, 1.75; 95% CI 1.03 to 2.97, *p* = 0.004) in the cervical group than in the thoracic group.

In cases where anterior support was added, the subgroup analysis revealed a significantly shorter ORT (WMD, −51.92; 95% CI −80.47 to −23.36, *p* < 0.001), a lower EBL (WMD, −165.19; 95% CI −225.69 to −104.70, *p* < 0.001), a higher incidence of overall ASD (RR, 1.88; 95% CI 1.16 to 3.06, *p* = 0.010), a higher incidence of distal ASD (RR, 2.38; 95% CI 1.35 to 4.19, *p* = 0.003), and a better NRS-NP score at the final follow-up (WMD, −0.69; 95% CI −1.17 to −0.21, *p* = 0.005) in the cervical group than in the thoracic group.

#### 3.10.2. Subgroup Analysis Based on Indications of Surgery

For specific myelopathy/radiculopathy, the subgroup analysis revealed a significantly lower incidence of wound-related complications (RR, 0.55; 95% CI 0.31 to 1.00, *p* = 0.049), a shorter ORT (WMD, −43.03; 95% CI −60.43 to −25.62, *p* < 0.001), and a lower EBL (WMD, −140.80; 95% CI −194.11 to −87.48, *p* < 0.001) in the cervical group than in the thoracic group.

For general indications, including myelopathy, radiculopathy, amyotrophy, deformity, pseudarthrosis, and OPLL, the subgroup analysis revealed a significantly lower overall surgical complication rate (RR, 0.40; 95% CI 0.19 to 0.88, *p* = 0.022), a lower incidence of neurologic deficits (RR, 0.30; 95% CI 0.10 to 0.84, *p* = 0.023), a shorter ORT (WMD, −44.68; 95% CI −72.89 to −16.46, *p* = 0.002), a lower EBL (WMD, −141.11; 95% CI −216.70 to −65.53, *p* < 0.001), and a better NRS-NP score at the final follow-up (WMD, −0.61; 95% CI −1.07 to −0.16, *p* = 0.008), while it revealed a higher incidence of overall ASD (RR, 2.38; 95% CI 1.30 to 4.36, *p* = 0.005), distal ASD (RR, 3.50; 95% CI 1.59 to 7.68, *p* = 0.002), overall hardware failure (RR, 1.53; 95% CI 1.04 to 2.27, *p* = 0.032), hardware failure of LIV (RR, 1.75; 95% CI 1.11 to 2.76, *p* = 0.016), and DJK (RR, 4.39; 95% CI 1.01 to 19.12, *p* = 0.049) in the cervical group than in the thoracic group.

### 3.11. Sensitivity Analyses

The sensitivity analyses indicated that the additional omission of any study would not significantly affect the results, which verified the stability of the data and rationale of the analyses.

### 3.12. Meta-Regression

The results of meta-regression did not suggest any association between each outcome and follow-up duration (*p* > 0.05) or age (*p* > 0.05).

## 4. Discussion

PCF is commonly performed for the treatment of degenerative cervical spine disease. However, surgeons often debate if LIV should be extended beyond the CTJ. The biomechanical characteristics of the CTJ are unique because this region is the transition between the mobile and lordotic cervical spine and the rigid and kyphotic thoracic spine [7]. Although several meta-analyses have been conducted to investigate the advantages and drawbacks of PCF construct crossing the CTJ, conflicting results have been reported [15,16,17,18]. Furthermore, potential sources of heterogeneity were introduced into these studies, but these factors (e.g., combination of anterior support in addition to PCF or indications for surgery) were not included in the subgroup analysis due to the limited number of studies. Therefore, the conclusions of previous meta-analyses may not have been rigorous enough, and this topic remains controversial.

The current study revealed no significant differences in the incidence of pseudarthrosis, DJK, surgical complications, systemic complications, reoperation rates, LOS, and radiographic outcomes when comparing the cervical and thoracic groups. The ORT was reasonably shorter and the EBL was lower when the PCF construct did not cross the CTJ. The cervical group was associated with a lower incidence of wound-related complications and a better NRS-NP score at the final follow-up; however, this group showed a higher incidence of overall ASD, distal ASD, overall hardware failure, and hardware failure of LIV. In the subgroup analysis, for the posterior approach alone, the cervical group presented with a lower incidence of wound-related complications and a better NDI score at the final follow-up, contrasted by a higher incidence of hardware failure of LIV and increased reoperation rate following mechanical complications. When anterior support was included, a higher incidence of overall ASD and distal ASD but a better NRS-NP score at the final follow-up were noted in the cervical group. For patients with specific myelopathy/radiculopathy, the benefits of crossing the CTJ were relatively limited. A higher incidence of wound-related complications as well as more surgical invasiveness were observed in the thoracic group. Alternatively, for general degenerative cervical spine diseases, the cervical group was associated with a lower incidence of overall surgical complications, a lower incidence of neurologic deficits, a better NRS-NP score at the final follow-up, but a higher incidence of overall ASD, distal ASD, overall hardware failure, hardware failure of LIV, and DJK. These findings had not been obtained in the previous meta-analyses [15,16,17,18].

ASD, hardware failure, pseudarthrosis, and reoperation for these mechanical complications are the main concerns when selecting the LIV for PCF. Biomechanical research has shown that lower cervical spinal fusion could significantly increase the range of motion and intradiscal pressure of the CTJ, which would induce degeneration and ASD [52,53,54]. In addition, instrumentation ending at the CTJ provides another rigid lever arm in this already stressed region [18,42]. The disruptive posterior ligamentous tension band caused by the posterior approach further destabilises the CTJ [8,52]. Instead, the thoracic spine has inherent stability and rigidity due to the sternum and ribs; therefore, PCF construct extending to the upper thoracic region may alleviate the increase in intradiscal pressure and stress at the CTJ [54]. This hypothesis was supported by the results of the current meta-analysis, which indicated that the incidence of both overall ASD (6.3% vs. 3.7%) and distal ASD (6.7% vs. 3.8%) were significantly lower in the thoracic group. Regarding the subgroup analysis, the benefits of crossing the CTJ in ASD were not observed in patients who underwent posterior procedures in isolation or had specific myelopathy/radiculopathy, but in those with anterior support or general indications. Additionally, if a high Pfirrmann grade of C7-T1 disc is detected, crossing the CTJ should also be considered.

Compared to lateral mass screws in lower cervical anchors, pedicle screws employed in the thoracic region could offer greater construct rigidity and stability, which is paramount for a stable mechanical environment [35]. In addition, a greater surface area for the fusion mass can be obtained by extension into the thoracic spine. Hence, solid fusion can be achieved, and the incidence of hardware failure would be reduced. In the current study, the incidence of pseudarthrosis was similar between the cervical and thoracic groups (2.7% vs. 4.0%), but the incidence of overall hardware failure (7.8% vs. 7.3%) and hardware failure of LIV (10.8% vs. 7.1%) were slightly higher in the cervical group. In subgroup analysis, for patients with general indications, LIV ending in the cervical region significantly increased the risk of hardware failure (11.6% vs. 11.1%), especially hardware failure of LIV (20.2% vs. 11.5%). Therefore, LIV terminated in the thoracic region could benefit patients who undergo PCF in terms of mechanical complications. Considering that patients with concurrent instability, ossification, or deformity usually face a high risk of ASD, hardware failure, or DJK, they usually require initial anterior-posterior surgery or posterior revisions for prior anterior surgery. Therefore, we advocate that crossing the CTJ should be considered for these patients and those with prior or concurrent anterior support [55,56].

In the current meta-analysis, the aetiology of reoperation was categorised into mechanical, surgical, and wound-related complications. Although there was no significant difference in the overall reoperation rate between the cervical and thoracic groups (9.6% vs. 10.1%), the reoperation rates for specific complication categories should be noted. As mentioned above, LIV terminating in the thoracic region could decrease the incidence of ASD and hardware failure, but the reoperation rate following mechanical complications was not statistically different between the groups (7.5% vs. 5.4%). Due to the relatively short follow-up duration (34.68 ± 14.85 months in the cervical group and 30.08 ± 17.40 months in the thoracic group), some patients with diagnosis of ASD or hardware failure may have been just at a high risk of reoperation, but there was no need for reoperation immediately at that time. However, the question remains if over time the shorter constructs would begin to fail and require revision at a rate significantly higher than the thoracic group. Therefore, when focusing on the outcomes and reoperations following PCF, follow-up with a longer term (5 years) is necessary.

Increased morbidity due to higher EBL and longer ORT was inevitable when the LIV was terminated in the thoracic region, with increasing risk of surgical site infection and wound dehiscence [10,48]. In this study, the incidence (2.9% vs. 5.9%) and reoperation rates (2.3% vs. 5.0%) of wound-related complications were significantly higher in the thoracic group. A more invasive operation may not be necessary for patients undergoing solely posterior procedures for specific myelopathy/radiculopathy, especially in the elderly population because its impact on wound-related complications is more severe for them. Therefore, surgeons should weigh the pros and cons before deciding on the LIV selection.

In contrast to previous studies, significant differences in PROs were detected in the current study [12,18]. The cervical group presented with a better NRS-NP score at the final follow-up, mainly for patients with concurrent instability, ossification, deformity, or a combination of anterior approach surgeries. For patients who underwent a solely posterior procedure, a better NDI score at the final follow-up was observed in the absence of CTJ crossing. This effect may be attributed to the less surgical invasiveness and the lower incidence of wound-related complications. However, cervical sagittal alignment is another critical determinant of PROs after PCF [57]. Several studies have shown that postoperative C2-7 cSVA negatively affects HRQoL in patients with degenerative cervical spine disease [58,59]. Owing to the biomechanical characteristics of the CTJ, the LIV terminating in this region could cause a compensatory increase in C2-7 cSVA and T1 slope. In the current study, although there was no significant difference in radiographic outcomes between the groups, extending the construct crossing the CTJ is still recommended for cases with preoperative cervical malalignment (C2-7 cSVA ≥ 40 mm), kyphotic deformity, or spondylolisthesis.

## 5. Limitations

This study has several limitations. Firstly, although subgroup analysis was performed, the inherent heterogeneity of the surgical techniques and indications could not be completely diminished. Moreover, most of the included studies only categorised the recruited patients according to the relationship between the LIV and the CTJ (e.g., cervical group or thoracic group) but not the specific LIV level (e.g., C6 group, C7 group, T1 group, or T2 group). This is another source of heterogeneity in this meta-analysis. Further studies should focus on the effect of LIV at different specific levels on patient outcomes. Secondly, most studies lacked data on whether reoperation was within the same hospitalisation, incidence of readmission, and length of time passed to reoperation, which are important information for the estimation of the real reoperation rate. Thirdly, several outcomes such as PROs and radiographic parameters were unavailable in some of the included studies. The current pooled outcomes may not be reliable when additional results are reported in future studies. Fourthly, all included studies were retrospective cohort studies, indicating that the selection of LIV was mainly based on the surgeon’s experience and preference. Randomised controlled trials with a higher level of methodological quality should be conducted to obtain more convincing conclusions. Most importantly, bone quality, frailty, corticosteroid administration, diabetes mellitus, and nutrition status are privy to careful patient selection, which is related to surgical indication and exigency [60]. However, the data of these critical baseline factors were not available in the included studies. It should be noted that the current meta-analysis evaluated a range of outcomes, and not all results of this study were derived from the same cohorts or the same studies. Despite the theoretical strengths of meta-analysis, patient selection, specific features of their local population, unknown biases of the investigators conducting the study, unknown rates of loss to follow-up, different pre-existing pathology, preoperative health, and levels of surgery, etc., can all introduce further bias and reduce generalisability. Surgeons who refer to the findings of this study should incorporate a commensurate amount of caution and keep these limitations in mind.

## 6. Conclusions

LIV of PCF construct terminating in the thoracic region did not demonstrate significant differences in pseudarthrosis, DJK, surgical complications, reoperations, surgical invasiveness, LOS, PROs and radiographic outcomes. Construct crossing the CTJ was associated with a lower incidence of ASD and hardware failure but a higher incidence of wound-related complications and a small increase in qualitative neck pain, without difference in neck disability on the NDI. Based on the subgroup analysis for surgical techniques and indications, prophylactic crossing of the CTJ should be considered for patients with concurrent instability, ossification, deformity, or a combination of anterior approach surgeries. However, long-term follow-up outcomes and patient selection-related factors such as bone quality, frailty, and nutrition status should be addressed in further studies.

## Figures and Tables

**Table 1:** Quality assessment of studies according to Newcastle-Ottawa Scale (NOS).

Author	Year	Selection	Comparability	Exposure	Total Score
Choi [37]	2018	3	2	2	7
Huang [38]	2019	4	2	2	8
Kennamer [39]	2019	3	1	2	6
Lee [40]	2019	3	2	2	7
Chan [41]	2021	4	2	3	9
Fayed [42]	2020	3	2	3	8
Hines [43]	2021	4	2	2	8
Labrum [44]	2021	3	2	3	8
Okamoto [45]	2021	4	2	3	9
Scholz [46]	2021	3	1	2	6
Sun [47]	2021	4	2	2	8
Truumees [48]	2020	4	1	2	7
Morin [49]	2022	4	2	2	8
Cho [50]	2022	3	2	2	7
Couch [51]	2022	4	1	2	7

## Data Availability

The data can be obtained by contacting the corresponding authors.

## Supplementary Materials

The following supporting information can be downloaded at: https://www.mdpi.com/article/10.3390/jcm12082806/s1, Table S1: Characteristics of the included studies; Table S2: Summary of pooled outcomes.
