# Supplementary material for: Is It Necessary to Cross the Cervicothoracic Junction in Posterior Cervical Decompression and Fusion for Multilevel Degenerative Cervical Spine Disease? A Systematic Review and Meta-Analysis
Source: J Clin Med. 2023 Apr 11;12(8):2806. doi: 10.3390/jcm12082806 (PMC10144726; doi:10.3390/jcm12082806)
Supplement: Supplementary file 1 [file jcm-12-02806-s001.zip › jcm-2234988-supplementary.pdf]

Table S1. Characteristics of the included studies

Authors	Year	Study Design	Level of Evidence	Group	Sample Size	Fusion Level	Age (years)	Sex (M/F)	BMI (kg/m²)	Active Smoker	Previous Surgery	Combination of AS	Diagnosis	FU (month)
Choi	2018	RCS	III	Cervical	29	4.1 ± 0.7	63.5 ± 10.9	13/16	NA	NA	NA	29/29	Myelopathy	43.1 ± 10.0
				Thoracic	21	5.3 ± 1.3	61.6 ± 9.5	5/16				21/21	Radiculopathy	35.4 ± 11.3
Huang	2019	RCS	III	Cervical	65	3.5 ± 0.8	62.4 ± 10.7	23/42	NA	9/65	7/65	NA	Myelopathy	24.0 ± 19.7
				Thoracic	64	5.8 ± 2.0	63.6 ± 12.6	43/21		8/64	15/64		Radiculopathy	29.7 ± 21.2
Kennamer	2019	RCS	III	Cervical	176	3.7 ± 0.7	65.2 ± 10.3	97/79	29.9 ± 5.1	17/176	NA	29/176	Stenosis<br>Myelopathy<br>Pseudarthrosis<br>Deformity	51.8 ± 16.6
				Thoracic	45	5.1 ± 1.2	66.4 ± 10.7	27/18	29.7 ± 6.0	8/45		13/45		53.9 ± 20.1
Lee	2019	RCS	III	Cervical	25	5.4 ± 0.6	61.0 ± 10.9	15/10	NA	NA	NA	NA	Myelopathy	38.1 ± 15.2
				Thoracic	21	7.3 ± 1.7	65.3 ± 9.9	12/9						38.9 ± 22.7
Fayed	2020	RCS	III	Cervical	79	NA	64.2 ± 9.4	52/27	29.0 ± 6.3	NA	NA	9/79	General degenerative disease	18.1 ± 13.9
				Thoracic	70		64.6 ± 11.6	29/41	28.3 ± 6.3			13/70		13.1 ± 9.8
Truumees	2020	RCS	III	Cervical	168	4.5 ± 1.0	62.0 ± 11.0	102/66	28.8 ± 6.7	41/168	NA	13/168	General degenerative disease	≥ 24.0
				Thoracic	96	6.8 ± 1.3	65.0 ± 11.0	53/43	28.9 ± 5.3	19/96		13/96		≥ 24.0
Chan	2021	RCS	III	Cervical	33	2.9 ± 0.7	59.1 ± 11.4	18/15	28.3 ± 4.8	6/33	1/33	NA	Myelopathy	≥ 12.0
				Thoracic	46	4.5 ± 1.2	63.7 ± 10.8	26/20	28.5 ± 6.3	7/46	1/46			≥ 12.0
Hines	2021	RCS	III	Cervical	185	4.6 ± 0.5	64.3 ± 11.6	NA	NA	35/185	NA	NA	Myelopathy	24.0 ± 25.9
				Thoracic	184	6.0 ± 0.1	63.0 ± 9.9			85/184				27.1 ± 37.1
Labrum	2021	RCS	III	Cervical	36	3.2 ± 1.1	59.2 ± 9.3	20/16	30.7 ± 7.9	8/36	NA	NA	Myelopathy<br>Radiculopathy<br>Pseudoarthrosis	36.3
				Thoracic	70	5.1 ± 1.5	61.3 ± 11.4	40/30	29.4 ± 6.2	17/70				31.7
Okamoto	2021	RCS	III	Cervical	43	5.3 ± 0.5	64.8 ± 12.4	29/14	26.0 ± 5.9	NA	7/43	NA	Myelopathy<br>Amyotrophy<br>OPLL	28.0 ± 14.8
				Thoracic	30	7.1 ± 0.8	62.5 ± 12.9	21/9	26.1 ± 6.9		6/30			30.5 ± 14.7
Scholz	2021	RCS	III	Cervical	20	4.8 ± 0.7	63.9 ± 9.6	17/3	NA	7/20	5/20	4/20	Myelopathy	124.6 ± 10.6
				Thoracic	38	6.3 ± 0.7	64.1 ± 10.4	26/12		14/38	10/38	10/38	OPLL	58.2 ± 15.7
Sun	2021	RCS	IV	Cervical	30	4.4 ± 0.7	60.8 ± 9.7	17/13	NA	NA	NA	NA	OPLL	15.4 ± 4.9
				Thoracic	6	6.8 ± 0.8	64.3 ± 8.1	4/2						17.0 ± 5.9
Cho	2022	RCS	III	Cervical	36	3.9 ± 0.9	55.8 ± 10.3	20/16	NA	NA	10/36	2/36	Myelopathy<br>Radiculopathy	≥ 24.0
				Thoracic	53	7.0 ± 2.7	56.6 ± 9.0	26/27			8/53	8/53		≥ 24.0

Couch	2022	RCS	III	Cervical<br>Thoracic	137<br>67	5.0 ± 0.3<br>6.4 ± 1.0	60.0 ± 10.2<br>65.7 ± 10.7	66/71<br>26/41	31.2 ± 6.7<br>30.0 ± 5.7	38/137<br>16/67	29/137<br>24/67	29/137<br>24/67	Myelopathy	33.1 ± 16.9<br>30.6 ± 15.5
Morin	2022	RCS	III	Cervical<br>Thoracic	101<br>97	4.5 ± 0.8<br>6.2 ± 1.0	62.3 ± 10.3<br>64.5 ± 9.2	75/26<br>51/46	29.4 ± 5.6<br>28.7 ± 5.9	19/101<br>27/97	NA	NA	Myelopathy	≥ 24.0<br>≥ 24.0

BMI indicates body mass index; AS, anterior support; FU, follow-up; RCS, retrospective cohort study; OPLL, posterior longitudinal ligament; NA, not available;

Table S2. Summary of pooled outcomes

Outcomes	Included Studies	Cervical (n)	Thoracic (n)	WMD or RR	95% CI		*P effect*	Heterogeneity	
								I^2	*P*
**Mechanical Complications**									
**Overall ASD**	12	1079	860	1.87	1.27	2.76	0.001	11.7%	0.330
Surgical technique subgroup									
No Combination of AS	6	463	491	1.85	0.97	3.54	0.062	0.0%	0.477
Combination of AS	6	616	369	1.88	1.16	3.06	0.010	37.2%	0.158
Surgical indication subgroup									
Specific	6	557	551	1.53	0.92	2.54	0.098	39.4%	0.143
General	6	522	349	2.38	1.30	4.36	0.005	0.0%	0.566
**Distal ASD**	8	642	555	2.18	1.36	3.51	0.001	21.5%	0.259
Surgical technique subgroup									
No Combination of AS	3	202	231	1.74	0.73	4.17	0.214	28.9%	0.245
Combination of AS	5	440	324	2.38	1.35	4.19	0.003	35.4%	0.185
Surgical indication subgroup									
Specific	4	339	281	1.47	0.81	2.69	0.206	56.0%	0.078
General	4	303	274	3.50	1.59	7.68	0.002	0.0%	0.891
**Proximal ASD**	4	382	298	1.18	0.34	4.13	0.799	0.0%	0.677
Surgical technique subgroup									
No Combination of AS	2	216	137	1.46	0.25	8.60	0.675	33.0%	0.222
Combination of AS	2	166	161	0.94	0.15	5.68	0.944	0.0%	0.958
**Overall hardware failure**	9	614	451	1.48	1.02	2.15	0.040	0.0%	0.779
Surgical technique subgroup									
No Combination of AS	5	202	231	1.53	0.97	2.42	0.066	0.0%	0.482
Combination of AS	4	412	220	1.37	0.71	2.62	0.349	0.0%	0.733
Surgical indication subgroup									
Specific	4	260	198	1.23	0.41	3.67	0.710	2.5%	0.380
General	5	354	253	1.53	1.04	2.27	0.032	0.0%	0.849
**Hardware failure of LIV**	6	380	339	1.89	1.21	2.95	0.005	0.0%	0.737
Surgical technique subgroup									
No Combination of AS	3	144	164	2.28	1.25	4.15	0.007	0.0%	0.637
Combination of AS	3	236	175	1.41	0.73	2.75	0.309	0.0%	0.569
Surgical indication subgroup									
Specific	2	202	131	4.37	0.56	33.89	0.159	0.0%	0.631
General	4	178	208	1.75	1.11	2.76	0.016	0.0%	0.592
**Pseudarthrosis**	8	839	599	0.72	0.41	1.25	0.237	0.0%	0.466
Surgical technique subgroup									
No Combination of AS	4	279	321	0.75	0.33	1.71	0.496	0.0%	0.726
Combination of AS	4	560	278	0.69	0.33	1.44	0.318	42.6%	0.156
Surgical indication subgroup									
Specific	4	380	318	0.85	0.35	2.11	0.733	0.0%	0.611
General	4	459	281	0.63	0.32	1.27	0.200	32.2%	0.219
**DJK**	6	517	348	1.57	0.60	4.08	0.358	11.9%	0.339

Surgical technique subgroup									
No Combination of AS	3	94	137	0.58	0.15	2.29	0.441	30.9%	0.235
Combination of AS	3	423	211	4.15	0.79	21.69	0.092	0.0%	0.694
Surgical indication subgroup									
Specific	2	58	67	0.22	0.03	1.77	0.153	0.0%	0.820
General	4	459	281	4.39	1.01	19.12	0.049	0.0%	0.853
**Surgical complications**									
**Overall**	8	646	508	0.66	0.40	1.10	0.113	23.8%	0.239
Surgical technique subgroup									
No Combination of AS	4	242	237	0.67	0.38	1.20	0.176	44.8%	0.143
Combination of AS	4	404	271	0.64	0.22	1.83	0.406	20.4%	0.288
Surgical indication subgroup									
Specific	4	336	274	1.04	0.51	2.10	0.915	0.0%	0.729
General	4	310	234	0.40	0.19	0.88	0.022	33.4%	0.212
**Epidural haematoma**	5	492	327	1.07	0.34	3.32	0.907	0.0%	0.614
Surgical technique subgroup									
No Combination of AS	2	108	94	0.84	0.13	5.40	0.854	18.2%	0.269
Combination of AS	3	384	233	1.22	0.29	5.09	0.789	0.0%	0.492
Surgical indication subgroup									
Specific	2	202	131	1.52	0.24	9.60	0.658	0.0%	0.585
General	3	290	196	0.86	0.20	3.70	0.842	5.9%	0.346
**Dural tears**	5	329	274	0.80	0.31	2.07	0.647	0.0%	0.461
Surgical technique subgroup									
No Combination of AS	3	141	140	0.72	0.24	2.16	0.554	25.7%	0.260
Combination of AS	2	188	134	1.04	0.16	6.77	0.970	0.0%	0.346
Surgical indication subgroup									
Specific	2	98	110	1.25	0.31	5.12	0.753	1.6%	0.313
General	3	231	164	0.56	0.15	2.13	0.392	0.0%	0.406
**Neurologic deficits**	6	408	344	0.46	0.21	1.01	0.052	0.0%	0.714
Surgical technique subgroup									
No Combination of AS	3	141	140	0.58	0.24	1.43	0.239	0.0%	0.376
Combination of AS	3	267	204	0.24	0.04	1.35	0.105	0.0%	0.933
Surgical indication subgroup									
Specific	2	98	110	0.99	0.26	3.75	0.992	0.0%	0.419
General	4	310	234	0.30	0.10	0.84	0.023	0.0%	0.714
**Wound-related complications**	9	831	692	0.58	0.36	0.92	0.022	7.3%	0.374
Surgical technique subgroup									
No Combination of AS	5	427	421	0.52	0.29	0.93	0.028	37.6%	0.171
Combination of AS	4	404	271	0.71	0.31	1.62	0.416	0.0%	0.589
Surgical indication subgroup									
Specific	5	521	458	0.55	0.31	1.00	0.049	30.2%	0.220
General	4	310	234	0.62	0.28	1.37	0.240	0.0%	0.421
**Systemic complications**	4	161	178	0.24	0.05	1.10	0.066	0.0%	0.999
Surgical indication subgroup									
Specific	2	98	110	0.23	0.03	1.94	0.178	0.0%	0.876
General	2	63	68	0.25	0.03	2.18	0.211	0.0%	0.955

**Reoperation**	11	1059	822	0.98	0.75	1.29	0.898	33.0%	0.135
Surgical technique subgroup									
No Combination of AS	6	463	491	1.02	0.70	1.50	0.914	48.1%	0.086
Combination of AS	5	596	331	0.94	0.64	1.39	0.762	21.3%	0.279
Surgical indication subgroup									
Specific	6	557	511	0.90	0.62	1.31	0.595	38.6%	0.148
General	5	502	311	1.09	0.73	1.62	0.678	40.3%	0.152
**Reoperation-Mechanical**	11	1059	822	1.38	0.98	1.95	0.063	29.8%	0.162
Surgical technique subgroup									
No Combination of AS	6	463	491	1.75	1.03	2.97	0.004	16.7%	0.306
Combination of AS	5	596	331	1.16	0.74	1.81	0.526	39.8%	0.156
Surgical indication subgroup									
Specific	6	557	511	1.51	0.88	2.58	0.136	16.0%	0.311
General	5	502	311	1.30	0.83	2.01	0.249	49.9%	0.092
**Reoperation-Surgical**	4	449	297	0.68	0.22	2.10	0.499	0.0%	0.560
Surgical indication subgroup									
Specific	2	202	131	0.98	0.16	5.94	0.983	0.0%	0.997
General	2	247	166	0.53	0.12	2.35	0.406	48.7%	0.163
**Reoperation-Wound**	7	768	624	0.55	0.32	0.96	0.034	0.0%	0.455
Surgical technique subgroup									
No Combination of AS	4	384	391	0.62	0.34	1.13	0.120	36.7%	0.192
Combination of AS	3	384	233	0.32	0.08	1.24	0.098	0.0%	0.938
Surgical indication subgroup									
Specific	5	521	458	0.59	0.33	1.04	0.070	24.9%	0.255
General	2	247	166	0.36	0.07	1.91	0.228	0.0%	0.799
**ORT**	10	611	570	-43.47	-59.42	-27.52	< 0.001	75.3%	< 0.001
Surgical technique subgroup									
No Combination of AS	6	308	313	-38.29	-57.84	-18.75	< 0.001	76.9%	0.001
Combination of AS	4	303	257	-51.92	-80.47	-23.36	< 0.001	73.3%	0.010
Surgical indication subgroup									
Specific	4	235	260	-43.03	-60.43	-25.62	< 0.001	63.9%	0.040
General	6	376	310	-44.68	-72.89	-16.46	0.002	82.1%	< 0.001
**EBL**	11	721	740	-143.77	-185.90	-101.63	< 0.001	59.4%	0.006
Surgical technique subgroup									
No Combination of AS	7	418	483	-130.25	-186.65	-73.85	< 0.001	54.7%	0.039
Combination of AS	4	303	257	-165.19	-225.69	-104.70	< 0.001	49.9%	0.112
Surgical indication subgroup									
Specific	5	345	430	-140.80	-194.11	-87.48	< 0.001	62.5%	0.030
General	6	376	310	-141.11	-216.70	-65.53	< 0.001	55.0%	0.049
**LOS**	7	621	596	-0.40	-1.28	0.48	0.375	81.0%	< 0.001
Surgical technique subgroup									
No Combination of AS	4	354	392	-0.18	-1.19	0.83	0.730	83.3%	< 0.001
Combination of AS	3	267	204	-0.86	-3.50	1.78	0.523	85.3%	0.001
Surgical indication subgroup									
Specific	3	318	322	-0.37	-1.61	0.87	0.556	78.8%	0.009
General	4	303	274	-0.49	-2.16	1.19	0.569	81.8%	0.001
**PROs**									

**NDI at final Follow-up**	8	413	278	-1.63	-4.50	1.23	0.264	77.5%	< 0.001
Surgical technique subgroup									
No Combination of AS	5	194	159	-3.60	-6.35	-0.85	0.010	72.0%	0.006
Combination of AS	3	219	119	3.44	-1.95	8.83	0.211	37.6%	0.202
Surgical indication subgroup									
Specific	4	170	185	-2.06	-7.01	2.89	0.415	84.8%	< 0.001
General	4	243	93	-0.85	-3.20	1.51	0.482	31.5%	0.223
**NRS-NP at final Follow-up**	6	327	268	-0.58	-0.93	-0.23	0.001	0.0%	0.470
Surgical technique subgroup									
No Combination of AS	4	151	151	-0.45	-0.97	0.08	0.093	26.2%	0.255
Combination of AS	2	176	117	-0.69	-1.17	-0.21	0.005	0.0%	0.780
Surgical indication subgroup									
Specific	3	121	130	-0.53	-1.09	0.03	0.064	42.0%	0.178
General	3	206	138	-0.61	-1.07	-0.16	0.008	0.0%	0.584
**NRS-AP at final Follow-up**	4	128	151	-0.39	-0.86	0.08	0.106	0.1%	0.391
**JOA score at final Follow-up**	5	139	151	0.05	-0.50	0.59	0.864	0.0%	0.636
Surgical indication subgroup									
Specific	3	101	108	0.28	-0.39	0.95	0.406	0.0%	0.849
General	2	38	43	-0.41	-1.35	0.52	0.386	0.0%	0.369
**Radiographic outcome**									
**C2-7 CL at final Follow-up**	6	301	160	0.36	-1.46	2.18	0.699	26.0%	0.239
Surgical technique subgroup									
No Combination of AS	4	131	103	0.51	-1.75	2.78	0.657	0.0%	0.432
Combination of AS	2	170	57	0.08	-3.00	3.15	0.962	74.7%	0.047
Surgical indication subgroup									
Specific	3	87	88	-1.20	-3.65	1.26	0.339	38.5%	0.197
General	3	214	72	2.28	-0.45	5.00	0.101	0.0%	0.972
**C2-7 cSVA at final Follow-up**	6	301	160	-0.56	-3.75	2.63	0.731	0.0%	0.543
Surgical technique subgroup									
No Combination of AS	4	131	103	-2.16	-6.20	1.89	0.296	0.0%	0.874
Combination of AS	2	170	57	2.05	-3.12	7.22	0.437	43.6%	0.183
Surgical indication subgroup									
Specific	3	87	88	-0.21	-4.61	4.18	0.925	47.9%	0.147
General	3	214	72	-0.94	-5.57	3.68	0.689	0.0%	0.925
**T1 Slope at final Follow-up**	3	101	97	-1.94	-4.72	0.83	0.170	0.0%	0.912

ASD indicates adjacent segment disease; LIV, lower instrumented vertebra; DJK, distal junctional kyphosis; Reoperation-Mechanical, reoperation rate following mechanical complications; Reoperation-Surgical, reoperation rate following surgical complications; Reoperation-Wound, reoperation rate following wound-related complications; ORT, operating time; EBL, estimated blood loss; LOS, length of hospital stay; PROs, patient-reported outcomes; NDI, Neck Disability Index; NRS-NP, numeric rating scale for neck pain; NRS-AP, numeric rating scale for arm pain; JOA, Japanese Orthopaedic Association; CL, cervical lordosis; cSVA, cervical sagittal vertical axis; AS, anterior support; WMD, weighted mean difference; RR, risk ratio; CI, confidence interval
